# Structural and Hormonal Changes in Reproductive-Age Women Post-COVID-19: A Cross-Sectional Ultrasound and Biochemical Study

**DOI:** 10.3390/diagnostics15121536

**Published:** 2025-06-17

**Authors:** Sandugash Yerkenova, Vyacheslav Lokshin, Almagul Kurmanova, Sharapat Moiynbayeva, Galiya Alikeyeva, Gulnara Kalibekova, Tamara Abdirova, Zamira Zhantenova, Kuralay Shaikova, Alla Mireeva, Aknur Turgumbayeva

**Affiliations:** 1 Department of Science and Consulting, Kazakhstan Medical University “Higher School of Public Health”, Almaty 050044, Kazakhstan; sanduka-85@mail.ru (S.Y.);; 2 International Clinical Center of Reproductology “PERSONA”, Almaty 050060, Kazakhstan; v_lokshin@persona-ivf.kz; 3 Faculty of Medicine and Healthcare, Al-Farabi Kazakh National University, Almaty 050040, Kazakhstan; 4 Department of Public Health, Asfendiyarov Kazakh National Medical University, Almaty 050012, Kazakhstan

**Keywords:** COVID-19, women’s health, reproductive health, endocrine disruption, ovarian function, post-COVID-19, pelvic ultrasound, AMH, estradiol

## Abstract

**Background/Objectives:** The long-term impact of COVID-19 on female reproductive health remains poorly understood. This study aimed to assess structural and endocrine alterations in women of reproductive age who had recovered from SARS-CoV-2 infection compared to uninfected controls. **Materials and Methods:** A total of 150 women aged 18–45 years were enrolled in a comparative study: 75 with a confirmed history of COVID-19 and 75 without one. All participants underwent ultrasound examinations of their pelvic organs and mammary glands, along with laboratory assessment of reproductive hormones and inflammatory markers. **Results:** Structural abnormalities in the pelvic organs were observed in 53.5% of the post-COVID-19 group versus 12.0% of the control group (*p* < 0.001), with oophoritis showing a statistically significant association (OR = 11.38; 95% CI: 1.42–91.36; *p* = 0.009). Non-significant but elevated frequencies were also found for uterine fibroids and breast cysts. Biochemically, post-COVID-19 participants demonstrated higher serum ferritin, estradiol, and fibrinogen levels, along with lower TSH and AMH levels, suggesting potential endocrine disruption and persistent inflammation. **Conclusions:** Women with a history of COVID-19 may be at increased risk of developing structural and hormonal abnormalities, highlighting the importance of post-infection gynecological and endocrine monitoring. Further longitudinal studies are required to elucidate the long-term effects and underlying mechanisms of these alterations.

## 1. Introduction

The COVID-19 pandemic caused by the SARS-CoV-2 virus not only resulted in millions of cases and deaths worldwide but also exposed hidden vulnerabilities in reproductive healthcare, particularly for women [1]. While initial attention was focused on the respiratory system, it soon became evident that the infection has systemic effects on the body, including the endocrine, vascular, and reproductive systems [2,3,4].

COVID-19 causes inflammatory changes in the pelvic organs and mammary glands and changes in hormonal balance, resulting in the development of dysmenorrhea. This can persist for several months after recovery, which can potentially lead to effects on fertility, adverse perinatal outcomes, reduced quality of life, and the need for specialized medical care [5,6,7,8].

Of particular concern is the fact that most changes after COVID-19 in women are detected incidentally and at later stages, making it difficult to assess health status when gynecological diseases become more serious [9]. To date, it remains poorly understood how frequently such structural changes occur, what they are associated with, and what mechanisms underlie their development [6]. Data obtained through objective instrumental methods—such as ultrasound examination (US), laboratory diagnostics, and hormonal screening—are especially limited.

According to systematic reviews, COVID-19 may be accompanied by menstrual irregularities, decreased ovarian reserve, exacerbations of chronic inflammatory diseases, and fibrocystic mastopathy [10,11]. Some authors have reported an increase in the incidence of follicular cysts, uterine fibroids, and endometrial hyperplasia in women after COVID-19 [12,13,14,15]. One original study demonstrated a decrease in anti-Müllerian hormone (AMH) levels and an increase in follicle-stimulating hormone (FSH) levels in women who had COVID-19 [16]. A series of studies confirmed that a number of women with post-COVID-19 syndrome report persistent reproductive problems, including structural changes in the pelvic organs and breasts [3,16].

The focus of our study is the use of objective diagnostic methods, including ultrasound imaging and laboratory testing, to identify and analyze possible morphological changes in the pelvic organs and mammary glands of women who have recovered from COVID-19.

Despite growing interest, only a few studies have objectively assessed the long-term impact of COVID-19 on the female reproductive system using standardized diagnostic methods [10,11].

A review of the literature reveals the presence of some clinical observations based on subjective complaints in small, heterogeneous samples. It remains unknown whether the observed changes are temporary or permanent and to what extent they are associated with the severity of the disease, the presence of post-COVID-19 syndrome, or hormonal dysregulation [13,17].

All of the above determined the use of a standardized diagnostic protocol in the present study, including ultrasound imaging and laboratory assessments of hormonal and metabolic markers [18,19]. In addition, to increase scientific rigor, a control group that was matched for age and other relevant characteristics was included, which allowed for an objective comparison between women who recovered from COVID-19 and those who were never infected [20,21].

It is expected that the results of this study will help to clarify the spectrum of structural changes occurring in the pelvic organs and mammary glands in the post-COVID-19 period, establish a potential link between previous SARS-CoV-2 infection and reproductive disorders, and serve as a basis for developing recommendations for clinical monitoring, early detection, and the prevention of complications [22,23].

The present study aims not only to fill the gap in objective data on structural changes in the pelvic organs and mammary glands of women of reproductive age following SARS-CoV-2 infection but also to create a scientific basis for improving healthcare and developing rehabilitation strategies for women of reproductive age who have had COVID-19.

Therefore, the problem addressed in this study is the insufficient exploration of the long-term effects of COVID-19 in women, particularly in terms of structural abnormalities in organs that are critical for reproductive and overall health. The timely detection and evaluation of such changes are essential for developing clinical guidelines and planning post-COVID-19 rehabilitation programs.

## 2. Materials and Methods

### 2.1. Study Design

This article presents an original prospective study, the purpose of which was to conduct a comprehensive assessment of structural abnormalities of the pelvic organs and mammary glands in women of reproductive age who had recovered from COVID-19 in comparison with women without a history of the disease.

A cross-sectional study was conducted involving 150 women of reproductive age (18–45 years) in August 2024, divided into two groups. The main group (post-COVID-19) included 75 women who had recovered from PCR-confirmed SARS-CoV-2 infection between 2021 and 2022. The control group comprised 75 women without one.

All participants underwent standardized clinical and laboratory evaluation, including hormone testing, biochemical marker analyses, and ultrasound imaging of the pelvic organs and mammary glands.

### 2.2. Participant Recruitment

Post-COVID-19 group: Data were collected from 110 women who were hospitalized at the Infectious Diseases Hospital of Almaty according to the national protocol with moderate and severe laboratory-confirmed COVID-19 during 2021–2022. Of these, 75 agreed to participate in the full clinical and laboratory assessment.

Control group: A digital questionnaire was distributed via WhatsApp Messenger, yielding 1170 responses. After excluding individuals who reported a history of COVID-19, we randomly selected 75 women who met the inclusion criteria (18–45 years old, no chronic diseases, living in Almaty, and signed informed consent).

**Figure 1:**
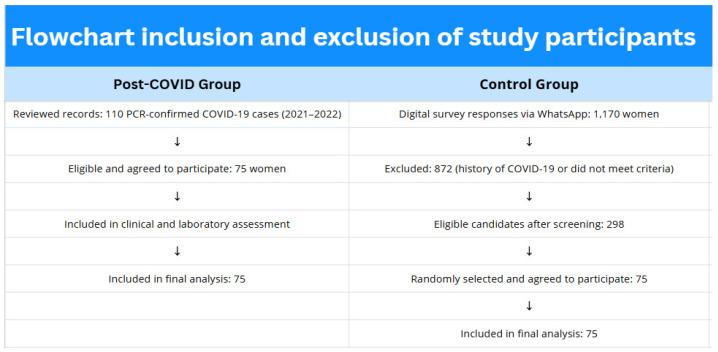
Flowchart illustrating the inclusion and exclusion of study participants in the post-COVID and control groups.

A summary of the inclusion and exclusion process is presented in Figure 1.

Given the constraints imposed by the pandemic and the need for rapid and cost-effective outreach, WhatsApp Messenger was selected as the primary platform for participant recruitment. This application is widely used in Kazakhstan and allows direct access to a diverse population of women of reproductive age residing in Almaty. The method enabled efficient distribution of the digital screening questionnaire while maintaining participant anonymity at the initial stage. To minimize selection bias, all respondents were screened based on pre-defined inclusion and exclusion criteria. Only those who explicitly consented to participate and met the eligibility requirements were contacted for further clinical evaluation.

### 2.3. Ethical Approval

All participants provided written informed consent prior to their inclusion. The study protocol was approved by the Local Ethics Committee of the Kazakhstan Medical University “Higher School of Public Health” (Protocol Number: IRB-253-2024).

### 2.4. Ultrasound Examinations

All participants underwent standardized ultrasound examinations of the pelvic organs and mammary glands using a Samsung WS80A, Samsung Medison, Seoul, Korea diagnostic ultrasound system. The scans were performed by certified sonographers in a clinical setting using a 3.5 MHz transabdominal probe for pelvic imaging and a 7.5–10 MHz linear probe for breast imaging. All ultrasound procedures followed international protocols and classification systems to ensure diagnostic consistency and reproducibility.

Pelvic scan: Transabdominal scans were performed with participants maintaining a full bladder to optimize visualization. The assessments included evaluations of the uterine size and position, endometrial thickness, ovarian morphology, and presence of structural abnormalities such as ovarian cysts, uterine fibroids, pelvic varicosities, and suspected endometriosis. The classification of abnormalities was based on the International Federation of Gynecology and Obstetrics (FIGO) system for uterine disorders, and the International Ovarian Tumor Analysis (IOTA) simple rules were applied where relevant.

The diagnosis of oophoritis was based on ultrasound criteria described in the literature for non-acute ovarian inflammation [24]. Specifically, the sonographic findings included enlarged ovary (>10 cm^3^), increased stromal echogenicity, peripheral displacement of follicles, and the presence of mild periadnexal fluid without adnexal torsion or complex cysts. All cases were evaluated by certified sonographers and confirmed by a second reviewer. Clinical symptoms such as pelvic discomfort or cycle irregularity were noted but not required for the ultrasound-based classification.

Breast scan: Bilateral breast ultrasound was conducted in a supine oblique position using a high-frequency linear probe. Structural changes such as simple or complex cysts, fibrocystic changes, and solid masses were evaluated and categorized according to the Breast Imaging Reporting and Data System (BI-RADS) developed by the American College of Radiology. Suspicious findings were documented in detail, and BI-RADS category 3 or above was considered clinically relevant.

Although Doppler ultrasound is a valuable tool in gynecology for assessing vascularization, ovarian torsion, and endometrial perfusion, it was not included in this study, as the primary objective was to evaluate structural (anatomical) rather than hemodynamic abnormalities. According to Nguyen P.N. and Nguyen V.T. (2023), Doppler modalities enhance diagnostic sensitivity in specific clinical scenarios, especially for suspected malignancy or vascular disorders [24]. Future studies should consider incorporating Doppler ultrasound to complement structural assessments with functional insights.

### 2.5. Laboratory Assessments

Blood samples were collected from all participants under standardized conditions by trained medical staff. For the control group, venous blood was drawn on days 3 to 7 of the menstrual cycle to ensure hormonal stability; in the post-COVID-19 group, sampling was performed irrespective of cycle phase due to the menstrual irregularities reported. All laboratory analyses were carried out at the “Sapa” Clinical Diagnostic Laboratory (Almaty, Kazakhstan), a certified and ISO-compliant facility accredited for clinical biochemical and immunological diagnostics. The following biomarkers were analyzed: reproductive hormones including estradiol (pg/mL), progesterone (ng/mL), prolactin (ng/mL), and anti-Müllerian hormone (AMH, ng/mL), measured using a chemiluminescent immunoassay (CLIA, Beckman Coulter Access 2); thyroid-stimulating hormone (TSH, mIU/L), measured via an enzyme-linked immunosorbent assay (ELISA, DRG Instruments GmbH); and inflammatory and coagulation markers, including ferritin (µg/L) and fibrinogen (g/L), analyzed using automated immunoturbidimetric and photometric assays (Abbott Architect, North Chicago, Illinois, USA and Sysmex CS-2500, Kobe, Japan). All tests were performed in duplicate to ensure their accuracy and internal validity, with inter-assay and intra-assay coefficients of variation maintained below 10%. The quality control procedures included daily verification using calibrators and control samples provided by the assay manufacturers.

### 2.6. Statistical Analysis

All statistical analyses were performed using SPSS software (version 20.0, IBM Corp., Armonk, NY, USA). Continuous variables were assessed for normality using the Shapiro–Wilk test. As most variables were non-normally distributed, data are presented as median values with interquartile ranges (Q1–Q3), and group comparisons were conducted using the non-parametric Mann–Whitney U-test. U-values < 338 indicate statistically significant differences at *p* < 0.05 (critical value for *n* = 75 per group). Categorical variables were compared using Pearson’s chi-square test or Fisher’s exact test, where appropriate.

## 3. Results

### 3.1. Baseline Characteristics

Before the outcomes were analyzed, the baseline demographic and clinical characteristics of the participants were compared between the post-COVID-19 and control groups. Table 1 summarizes the key parameters, including age, body mass index (BMI) [25], reproductive history, and gynecologic health status.

No statistically significant differences were observed between the groups in terms of age, BMI, marital status, or parity. However, a higher proportion of women in the post-COVID-19 group reported irregular menstrual cycles (36.0% vs. 20.0%, *p* = 0.035), which may reflect post-infection hormonal dysregulation.

### 3.2. Menstrual Irregularities

In addition to imaging and biochemical parameters, the participants’ menstrual cycle patterns were assessed during structured clinical interviews. Menstrual irregularities were defined as self-reported cycles shorter than 21 days, cycles longer than 35 days, or missed periods lasting more than one cycle, excluding cases explained by hormonal contraception or lactation.

Among the women in the post-COVID-19 group, 27 out of 75 participants (36.0%) reported menstrual irregularities, compared to 15 out of 75 women (20.0%) in the control group. This difference was statistically significant (χ^2^ = 4.53; *p* = 0.035), indicating a potential association between prior SARS-CoV-2 infection and disruption of the hypothalamic–pituitary–ovarian (HPO) axis. These findings are consistent with emerging clinical reports of post-COVID-19 menstrual dysfunction and highlight the importance of including menstrual health in post-infectious reproductive assessments.

### 3.3. Ultrasound Examination of the Pelvic Organs

The mean age of the participants in both groups was 33.5 ± 12.8 years. Among the 150 women assessed (75 with a confirmed history of COVID-19 and 75 controls), structural abnormalities in pelvic organs were significantly more prevalent in the post-COVID-19 group (53.5%) compared to the control group (12.0%, *p* < 0.001).

Ultrasound examination revealed that oophoritis was significantly more frequent in the post-COVID-19 group (13.3%) than in the controls (1.3%), with an odds ratio (OR) of 11.38 (95% confidence interval [CI]: 1.42–91.36, *p* = 0.009). Other abnormalities were also observed more commonly in the post-COVID-19 group: uterine fibroids (12.0% vs. 2.7%; OR = 4.98, 95% CI: 1.04–23.88; *p* = 0.056), ovarian cysts (10.7% vs. 4.0%; OR = 2.87, *p* = 0.209), pelvic varicose veins (9.3% vs. 2.7%; OR = 3.76, *p* = 0.166), and chronic endometriosis (6.7% vs. 1.3%; OR = 5.29, *p* = 0.209). However, these differences did not reach statistical significance, although the trend for uterine fibroids was borderline.

**Figure 2:**
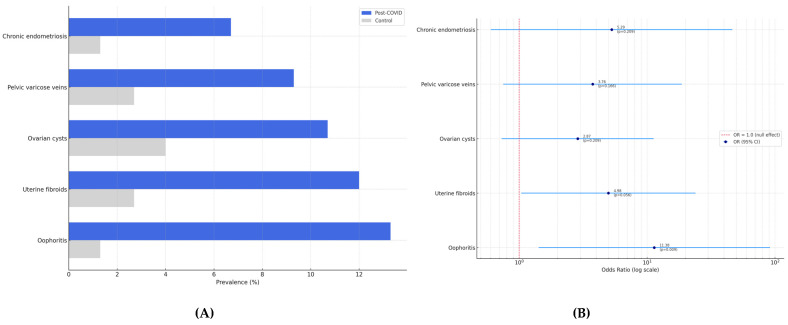
(**A**) Prevalence of pelvic structural abnormalities in women with and without prior COVID-19. (**B**) Odds ratios with 95% confidence intervals for the same conditions.

These findings are summarized in Table 2 and visualized in Figure 2A, which displays the comparative prevalence of abnormalities in each group, and Figure 2B, which shows the corresponding odds ratios with 95% confidence intervals.

### 3.4. Breast Ultrasound

In addition to pelvic organ abnormalities, this study evaluated potential structural changes in the breast tissue of women with and without a history of COVID-19. This was motivated by increasing clinical reports of post-infectious mastopathy and breast cyst formation following viral infections, including SARS-CoV-2.

Ultrasound assessment of the mammary glands revealed a higher frequency of structural abnormalities in the post-COVID-19 group. Specifically, breast cysts were detected in 10.7% of women with a history of COVID-19 compared to 2.7% in the control group (OR = 4.43; 95% CI: 0.91–21.53; *p* = 0.082). Fibrocystic breast disease was observed in 6.7% of post-COVID-19 participants versus 1.3% in controls (OR = 5.29; 95% CI: 0.60–46.38; *p* = 0.209). Although these differences did not reach statistical significance, they reflect a consistent trend similar to that in the pelvic findings.

A comparative summary of all structural abnormalities identified in both pelvic and breast tissues is presented in Table 3.

**Figure 3:**
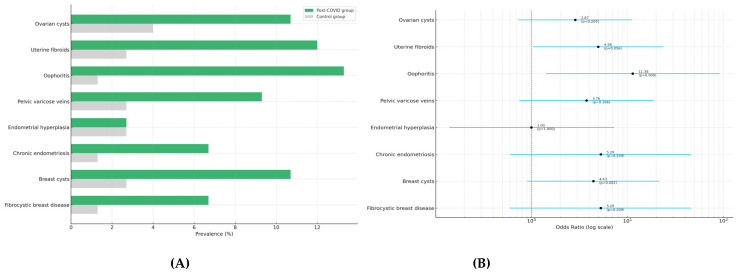
(**A**) Prevalence of pelvic and breast structural abnormalities in post-COVID-19 and control groups. (**B**) Odds ratios with 95% confidence intervals for the same conditions.

Visual representations are shown in Figure 3A (prevalence by group) and Figure 3B (odds ratios with 95% confidence intervals).

### 3.5. Biochemical and Hormonal Changes

In addition to structural abnormalities, we examined potential biochemical and hormonal alterations associated with COVID-19. A panel of reproductive hormones and inflammatory markers was analyzed in both groups to explore possible endocrine and metabolic effects of the virus.

A descriptive analysis of the hormonal and inflammatory markers revealed several notable differences between the post-COVID-19 and control groups (Table 4).

Women with a history of COVID-19 had higher levels of ferritin (median 420.0 μg/L vs. 311.0 μg/L) and estradiol (57.0 pg/mL vs. 11.2 pg/mL).

## 4. Discussion

This study aimed to investigate potential structural, hormonal, and inflammatory changes in women of reproductive age following COVID-19 infection. The main finding was a significantly higher prevalence of pelvic abnormalities in the post-COVID-19 group compared to the controls (53.5% vs. 12.0%; *p* < 0.001), with oophoritis being the only statistically significant individual abnormality (OR = 11.38; *p* = 0.009). Menstrual irregularities were also significantly more frequent in the post-COVID-19 group (36.0% vs. 20.0%; *p* = 0.035). These findings suggest a possible link between SARS-CoV-2 infection and subclinical reproductive pathology.

Other structural abnormalities—such as uterine fibroids, ovarian cysts, and breast cysts—were more prevalent in the post-COVID-19 group, although these differences did not reach statistical significance. Nonetheless, the consistent upward trends in these conditions may reflect a broader inflammatory or hormonal impact of COVID-19 and warrant further investigation.

The problem addressed herein is the insufficient study of the long-term consequences of COVID-19 in women, especially in terms of structural abnormalities in organs that are critical for reproductive and general health. The timely detection and assessment of such changes are important for the development of post-COVID-19 rehabilitation programs [26,27]. The hypothesis of this study is that women of reproductive age who have had COVID-19 have a higher frequency of morphological abnormalities in the pelvic organs and mammary glands compared to conditionally healthy women. This may be a consequence of systemic inflammation, microcirculatory disorders, and hormonal imbalance caused by the infection [28].

The hypothesis of a post-viral effect on reproductive organs is supported by multiple mechanisms, including inflammation, immune dysregulation, and direct endocrine interference [7,8,9,10,15]. Previous studies have described altered ovarian morphology and fibrocystic breast changes post-COVID-19 [6,16]. Regional epidemiological data from Kazakhstan also indicate an increase in breast pathology burden, which aligns with our findings.

From a biochemical perspective, the post-COVID-19 group exhibited elevated ferritin, estradiol, and fibrinogen levels, along with reduced TSH and AMH levels. These changes are consistent with known COVID-19-related systemic inflammation and endocrine disruption [29]. Fibrinogen elevation is particularly relevant due to its role in hypercoagulability and thrombotic complications [23]. Decreased TSH levels may indicate transient or long-term thyroid dysfunction in COVID-19 survivors.

Our study also confirmed a higher incidence of menstrual irregularities among women post-COVID-19, which supports recent population-based studies [12,29,30]. Women with severe COVID-19 symptoms were found to be more likely to have an earlier onset of menstrual irregularities [29]. These disruptions may result from hypothalamic–pituitary–ovarian axis dysregulation, direct viral effects on the ovaries, or persistent systemic inflammation [12,13,14,15,16].

The most robust finding—statistically and clinically—was the increased incidence of oophoritis in the post-COVID-19 group. This condition likely reflects localized ovarian inflammation and may be associated with diminished ovarian reserve. Similar trends were reported by Ermakova et al., who observed post-COVID-19 follicular depletion and hormonal imbalance [16].

These findings suggest that women of reproductive age who have recovered from COVID-19 may be at increased risk of developing structural abnormalities in both pelvic and breast tissues. While only oophoritis demonstrated a statistically significant association, the observed trends point toward a potentially greater inflammatory or hormonal burden in the post-COVID-19 group. These results highlight the importance of integrated gynecological and breast health surveillance in women after SARS-CoV-2 infection.

Based on our results, we recommend individualized post-COVID-19 monitoring for women of reproductive age, particularly those presenting with menstrual changes or pelvic discomfort. Ultrasound assessment and hormonal profiling may aid in the early detection of subclinical abnormalities. These findings also have implications for preconception care in COVID-19 survivors [18].

This study has several limitations. The relatively small sample size limited the statistical power for less common findings. The cross-sectional design precludes causal inferences. Furthermore, hormonal sampling in the post-COVID-19 group was not cycle-synchronized, introducing potential variability in the endocrine data. A limitation was that the type and dose of the COVID-19 vaccine were not studied.

In conclusion, our findings suggest that COVID-19 may have lasting effects on female reproductive and endocrine health. While only oophoritis and menstrual irregularities reached statistical significance, observed hormonal and structural trends highlight the need for continued investigation. Future longitudinal studies should aim to clarify causal mechanisms and guide the development of targeted clinical management strategies.

The novelty of this study lies in its integrated and objective evaluation of post-COVID-19 reproductive health using standardized ultrasound protocols and laboratory diagnostics. Unlike previous research, which has predominantly relied on subjective self-reports or small case series, this study employed a comparative design with matched controls and blinded evaluations. Furthermore, it is one of the first studies in Central Asia to document a statistically significant association between SARS-CoV-2 infection and oophoritis, alongside menstrual disruptions and endocrine shifts. These findings contribute valuable clinical evidence to the global understanding of long-term reproductive sequelae after COVID-19 and provide a rationale for implementing targeted follow-up protocols and preconception care strategies in post-COVID-19 populations.

## 5. Conclusions

This study has demonstrated that women of reproductive age who have recovered from COVID-19 may develop specific structural and endocrine alterations. Oophoritis was the only statistically significant abnormality identified on ultrasound, occurring markedly more often in the post-COVID-19 group. Menstrual irregularities were also significantly more common, supporting the hypothesis of post-infectious reproductive disruption.

Although other structural and hormonal changes—such as ovarian cysts, uterine fibroids, breast cysts, and altered AMH and TSH levels—did not reach statistical significance, they showed consistent upward trends in the post-COVID-19 group. These patterns may indicate a broader reproductive vulnerability that warrants further study.

Our findings emphasize the importance of routine gynecologic and hormonal assessment in women following SARS-CoV-2 infection. Larger, longitudinal studies are needed to confirm these associations, identify underlying mechanisms, and inform clinical guidelines for reproductive monitoring and care after COVID-19.

## Figures and Tables

**Table 1:** Baseline characteristics of the study population (*n* = 150).

Characteristic	Post-COVID-19 Group (*n* = 75)	Control Group (*n* = 75)	*p*-Value
Age, years (mean ± SD)	33.6 ± 5.2	32.9 ± 5.7	0.482
BMI, kg/m^2^ (mean ± SD)	24.8 ± 3.9	24.1 ± 3.5	0.293
Urban residence, *n* (%)	72 (96%)	74 (98.7%)	0.618
Married, *n* (%)	58 (77.3%)	60 (80.0%)	0.693
Nulliparous, *n* (%)	18 (24.0%)	21 (28.0%)	0.568
History of irregular menstruation, *n* (%)	27 (36.0%)	15 (20.0%)	0.035 *
History of gynecologic diseases, *n* (%)	19 (25.3%)	12 (16.0%)	0.164
Smoking, *n* (%)	6 (8.0%)	4 (5.3%)	0.515
COVID-19 vaccination, *n* (%)	47 (62.7%)	0 (excluded by design)	-

Note: Data are presented as the mean ± standard deviation (SD) for continuous variables and number (%) for categorical variables. Statistical significance assessed using Student’s *t*-test or the chi-square test, as appropriate. * *p* < 0.05 is considered statistically significant.

**Table 2:** Prevalence of pelvic structural abnormalities and association with prior COVID-19 infection. OR = odds ratio; CI = confidence interval. Significant values are marked in bold.

Indicators	Post-COVID-19 (%)	Control (%)	OR (95% CI)	*p*-Value
Presence of structural changes (%)	53.5	12.0	8.56 (3.85–19.06)	**<0.001** ^a^
Ovarian cysts (%)	10.7	4.0	2.87 (0.73–11.25)	0.209
Uterine fibroids (%)	12.0	2.7	4.98 (1.04–23.88)	0.056
**Oophoritis (%)**	**13.3**	**1.3**	**11.38 (1.42–91.36)**	**0.009** ^b^
Pelvic varicose veins (%) Endometriosis (%)	9.3 6.7	2.7 1.3	3.76 (0.75–18.72) 5.29 (0.60–46.38)	0.166 0.209

^a^—borderline; ^b^— statistically significant. OR = odds ratio; CI = confidence interval. Statistically significant results are shown in bold.

**Table 3:** Comparison of pelvic and breast structural abnormalities in post-COVID-19 and control groups (*n* = 75 per group).

Indicators	Post-COVID-19 (%)	Control (%)	OR (95% CI)	*p*-Value
Ovarian cysts	10.7	4.0	2.87 (0.73–11.25)	0.209
Uterine fibroids	12.0	2.7	4.98 (1.04–23.88)	0.056 ^a^
**Oophoritis**	**13.3**	**1.3**	**11.38 (1.42–91.36)**	**0.009** ^b^
Pelvic varicose veins	9.3	2.7	3.76 (0.75–18.72)	0.166
Endometrial hyperplasia	2.7	2.7	1.00 (0.14–7.29)	1.000
Chronic endometriosis	6.7	1.3	5.29 (0.60–46.38)	0.209
Breast cysts	10.7	2.7	4.43 (0.91–21.53)	0.082
Fibrocystic breast disease	6.7	1.3	5.29 (0.60–46.38)	0.209

^a^—borderline; ^b^—statistically significant. OR = odds ratio; CI = confidence interval. Statistically significant results are shown in bold.

**Table 4:** Comparison of hormone and biomarker levels between post-COVID and control groups (median ± IQR).

Marker	Post-COVID-19 Group (Median ± IQR)	Control Group (Median ± IQR)	*p*-Value
Fibrinogen (g/L)	3.05 ± 2.5–3.5	2.80 ± 2.3–3.1	<0.05
TSH (mIU/L)	3.30 ± 3.0–3.5	3.70 ± 3.5–4.0	>0.05
Ferritin (μg/L)	420.0 ± 350–490	311.0 ± 280–340	<0.05
Estradiol (pg/mL)	57.0 ± 50–65	11.2 ± 10–12	<0.05
Prolactin (ng/mL)	15.0 ± 12–18	15.0 ± 12–18	>0.05
Progesterone (ng/mL)	8.0 ± 6–10	10.0 ± 8–12	>0.05
AMH (ng/mL)	2.5 ± 2.0–3.0	3.0 ± 2.5–3.5	>0.05

Values are presented as the median ± interquartile range (IQR). TSH—thyroid-stimulating hormone; AMH—anti-Müllerian hormone.

## Data Availability

The data presented in this study are available from the corresponding author upon request. The data are not publicly available due to privacy and ethical issues.
